# Cell Adhesion-Mediated Actomyosin Assembly Regulates the Activity of Cubitus Interruptus for Hematopoietic Progenitor Maintenance in *Drosophila*

**DOI:** 10.1534/genetics.119.302209

**Published:** 2019-05-28

**Authors:** Shiv Kumar Sharma, Saikat Ghosh, Aarathy RavisundarJose Geetha, Sudip Mandal, Lolitika Mandal

**Affiliations:** * Developmental Genetics Laboratory, Department of Biological Sciences, Indian Institute of Science Education and Research (IISER), Mohali 140306, India; † Molecular Cell and Developmental Biology Laboratory, Department of Biological Sciences, Indian Institute of Science Education and Research (IISER), Mohali 140306, India

**Keywords:** DE-cadherin, actomyosin network, hematopoietic progenitors, *Drosophila* Hedgehog

## Abstract

The actomyosin network is involved in crucial cellular processes including morphogenesis, cell adhesion, apoptosis, proliferation, differentiation, and collective cell migration in *Drosophila*, *Caenorhabditis* *elegans*, and mammals. Here, we demonstrate that *Drosophila* larval blood stem-like progenitors require actomyosin activity for their maintenance. Genetic loss of the actomyosin network from progenitors caused a decline in their number. Likewise, the progenitor population increased upon sustained actomyosin activation via phosphorylation by Rho-associated kinase. We show that actomyosin positively regulates larval blood progenitors by controlling the maintenance factor Cubitus interruptus (Ci). Overexpression of the maintenance signal via a constitutively activated construct (ci.HA) failed to sustain Ci-155 in the absence of actomyosin components like Zipper (zip) and Squash (sqh), thus favoring protein kinase A (PKA)-independent regulation of Ci activity. Furthermore, we demonstrate that a change in cortical actomyosin assembly mediated by DE-cadherin modulates Ci activity, thereby determining progenitor status. Thus, loss of cell adhesion and downstream actomyosin activity results in desensitization of the progenitors to Hh signaling, leading to their differentiation. Our data reveal how cell adhesion and the actomyosin network cooperate to influence patterning, morphogenesis, and maintenance of the hematopoietic stem-like progenitor pool in the developing *Drosophila* hematopoietic organ.

STUDIES over the last decade have revealed remarkable similarities between *Drosophila* blood cell development and vertebrate hematopoiesis (Evans *et al.* 2003; Jung *et al.* 2005; Letourneau *et al.* 2016; Yu *et al.* 2018). Most of this work has focused on the larval blood-forming, multi-lobed organ known as the lymph gland. In third instar larvae, the anterior lobe of the lymph gland becomes organized into three distinct domains (Jung *et al.* 2005; Krzemień *et al.* 2010) (Figure 1, A and A’). The outer periphery (the cortical zone, CZ) consists of differentiated blood cells, while the core of the organ is populated by stem-like progenitors (medullary zone, MZ). Posterior to these two domains lies a cluster of cells that form the Posterior Signaling Center (PSC), which serves as the hematopoietic niche (Krzemień *et al.* 2007; Mandal *et al.* 2007; Baldeosingh *et al.* 2018) crucial for progenitor cell maintenance via Hedgehog (Hh) signaling (Mandal *et al.* 2007; Tokusumi *et al.* 2010; Baldeosingh *et al.* 2018). Although one report contests the role of the PSC/niche in blood progenitor maintenance (Benmimoun *et al.* 2015), a vast body of literature endorses the PSC’s instructive role in hematopoietic progenitor maintenance via Hh signaling (Mandal *et al.* 2007; Tokusumi *et al.* 2010, 2012, 2015; Mondal *et al.* 2011; Benmimoun *et al.* 2012; Lam *et al.* 2014; Grigorian *et al.* 2017; Hao and Jin 2017; Khadilkar *et al.* 2017; Baldeosingh *et al.* 2018; Banerjee *et al.* 2019). A direct readout of Hh signaling in the progenitors is the expression of the full-length Cubitus interruptus (Ci-155) (Motzny and Holmgren 1995), and progenitor-specific downregulation of Ci activation affects their maintenance (Mandal *et al.* 2007).

**Figure 1:**
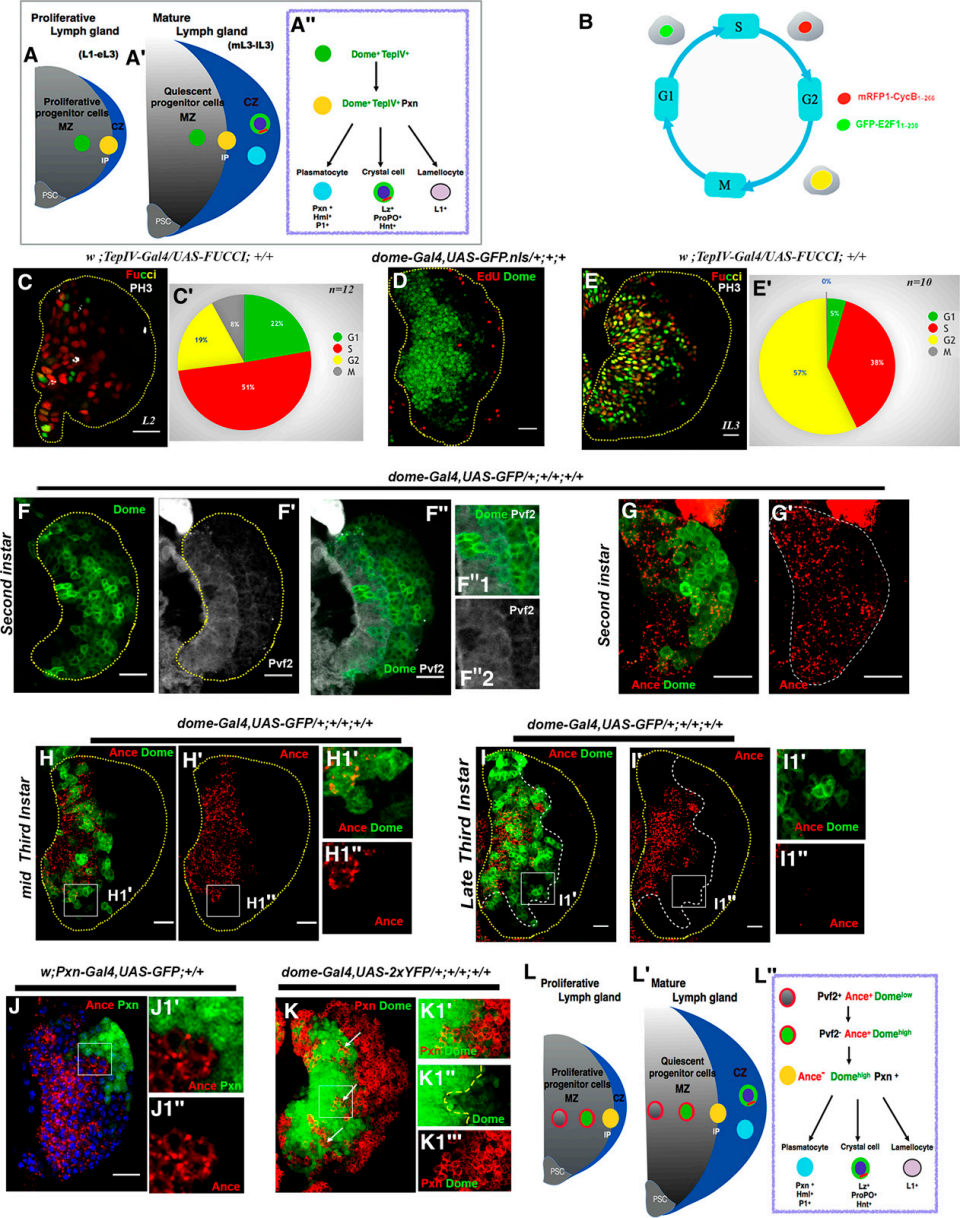
*Drosophila* hematopoietic progenitors are heterogeneous. The genotypes are mentioned on the top of the relevant panels. (A–A’’) Schematic representation of lymph gland in early (A) and late instar stages (A’). The hemocyte progenitor cells housed in the medullary zone (MZ) of the lymph gland are proliferative in early stages and quiescent in late larval stages. They can be identified by Domeless and TepIV expression. These cells upon maturation give rise to plasmatocytes, crystal cells, and lamellocytes (during infection), which then populate the peripheral region forming the cortical zone (CZ). An intermediate zone evolves in this process wherein the differentiating progenitors are low in *dome*-GFP and co-express Pxn (yellow (A’)). (A’’) *Drosophila* blood cells hierarchy in developing lymph gland. (B) The scheme is describing the Fly-FUCCI-fluorescent ubiquitination-based cell cycle indicator. This system uses two probes, the first of which is E2F moiety fused to GFP. Since Cdt2 degrades E2F during S, the GFP marks cells in G1, G2, and M phases of cell cycle only. The second probe coupled with this system is CycB moiety fused to mRFP. This moiety is susceptible to degradation by APC/C during the G1 phase, as an outcome of which the RFP tagged to it marks cells in S and those undergoing G2/mitosis in yellow. (C–E’) Cell cycle status reported by Fly-FUCCI using progenitor-specific GAL4: *TepIV-Gal4*. Early on (C) progenitors in the lymph gland are in S phase (red), while few of them are in G1 (green) and G2 (yellow). (D and E) In late third instar lymph gland, the progenitors (green) lack EdU (red)/PH3 ((E) gray) incorporation is paused in G2 (FUCCI: yellow). (C’ and E’) Quantitative analyses of the cell cycle of the early and late progenitors. (F and F’) Hemocyte progenitors are heterogeneous. In second instar larval lymph glands few cells near the dorsal vessel are low in GFP (F) that corresponds to Pvf2 lacZ (gray, F’ and F’’ and F’’1 and F’’2). (G and G’) Ance expression (red) in the early hematopoietic progenitor cells visualized by Dome GFP (*dome-Gal4*, *UAS-GFP*) in developing lymph glands. (H–I1’’) Ance expression (red) marks a restricted population of DomeGFP cells in midthird instar lymph gland. (H1’–I1’’) Higher magnification of selected areas in H and I show that cells in the periphery of MZ are low in DomeGFP and negative for Ance. (J–K) Expression of Ance (red) is restricted to MZ. Pxn GFP-positive differentiated cells are negative for Ance (J1’ and J1’’) but express low levels of *Dome* (K–K1’’’) near the periphery of the MZ. Co-localization of Pxn (red) and Dome-Gal4, *UAS-YFP* in third instar lymph gland efficiently marks the intermediate progenitors (IP, arrows in K). (L–L’’) A scheme based on above results describing the heterogeneous progenitors of MZ in the larval lymph gland. The yellow dotted line marks the whole of the lymph gland in all cases, while white marks the progenitors in G’ and I’. L1, eL3, mL3, and lL3 are early first instar, early, mid and late stages of third larval instar. The nuclei are marked with DAPI (blue) in J. See also Figure S1. Bar, 20 μm.

As the lymph gland grows, there is also an increase in the number of progenitors in the MZ that are no longer near the Hh-expressing niche. Studies have shown that at this developmental time point, signals arising from differentiating cells in the CZ collaborate with the PSC/niche-derived signal to evoke quiescence in the progenitors (Mondal *et al.* 2011).

Lineage analyses have confirmed the presence of a fourth domain in the lymph gland (between the MZ and CZ) that contains a rim of intermediate progenitor (IP) cells that initiate blood cell differentiation (Sinenko *et al.* 2009; Krzemień *et al.* 2010).

Although the zonation within the lymph gland is well-defined (Jung *et al.* 2005), how various spatial and temporal events regulate the patterning and zonation within the developing organ remains to be elucidated.

In this report, we address the spatiotemporal events occurring within the progenitors that lead to their differentiation via an in-depth characterization of known and novel genetic markers, including functional analyses in loss- and gain-of-function genetic backgrounds.

We have identified cell–cell adhesion and actomyosin activity as crucial players in maintaining progenitor homeostasis. The involvement of the actomyosin network in key cellular processes, including morphogenesis (Martin *et al.* 2009; Nie *et al.* 2014), cell adhesion, apoptosis, proliferation, differentiation, and collective cell migration, has been previously reported in *Drosophila*, *Caenorhabditis* *elegans*, and mammals (Vicente-Manzanares *et al.* 2009; Wozniak and Chen 2009; Cai *et al.* 2014; Munjal and Lecuit 2014; Rauskolb *et al.* 2014; Murrell *et al.* 2015). The intracellular actomyosin network, which consists of actin filaments, actin cross-linkers, and myosin motors, induces changes in cell shape that initiate various cell biological processes (Laevsky and Knecht 2003). Upstream of this cellular remodeling process are cell adhesion factors, *i.e.*, cadherin and integrin, which help to transmit the cellular tension to the cell cortex to facilitate tissue morphogenesis. Our study genetically links cell–cell adhesion and high actomyosin cytoskeleton activity with Ci level in a network that is essential for progenitor maintenance. Loss of both functions during development, specifically in the cells adjacent to the MZ progenitors, downregulates Ci-155 expression that eventually lead to the formation of intermediate progenitors. As a consequence, the IP cells differentiate.

That cell adhesion and activity of actomyosin components regulate morphogen-dependent patterning in the developing *Drosophila* lymph gland progenitors is the significant finding in our study.

## Materials and Methods

### Fly stocks

The fly stocks used were *antp-Gal4* (S. Cohen), *dome-Gal4* (U. Banerjee), *pcol-Gal4* (Crozatier M.), *ZCL1973 (X)* (Lynn Cooley), *Pvf2-LacZ* (M. A. Yoo), *hhF4-GFP* (R. A. Schulz), *UAS-HhGFP* (S. Mayor), *dome-MesoEBF2* (U. Banerjee), *Hml-GAL4.Δ* (S. Sinenko). *Pxn-Gal4*, *UAS-ance RNAi (BL36749)*, *UAS-zip RNAi (BL36727)*, UAS-*smo* RNAi (BL27037), UAS-smo.act (BL44621), *UAS-ci.HA (BL32569)*, *UAS-ROCK (BL6668)*, *UAS-FUCCI (BL55121)*, *UAS-mCD8RFP (BL27399)*, *UAS-shgRNAi (BL 32904)*, *Sqh^AX1^*; *SqhGFP (BL57144)*, *UAS-2xEGFP (BL6874)*, *Ance*^34Eb2^(BL3584), *Df(2L)b*^88f32^(BL3897), tub-*GAL80^ts^(BL7019)* on second chromosome, tub-*GAL80^ts^(BL7017)* on third chromosome, *hsFLP (BL8862)*, and *ay-GAL4 (BL3953)* are from Bloomington *Drosophila* Stock Center, Indiana University. *Pxn-YFP* (DGRC 11545) and *TepIV-Gal4* (DGRC 105442) are from *Drosophila* Genomic Research Center, Kyoto, Japan. *UAS-sqh RNAi* (VDRC, GD 7916) was obtained from Vienna *Drosophila* Resource Center.

All stocks and crosses were maintained at 25°, except for those used in RNAi and *GAL4/UAS* expression experiments. In those cases, crosses were maintained at 29°. For *GAL80^ts^* experiments, crosses were initially maintained at 18° (permissive temperature) for 7 days and 2 hr (equivalent to 60 hr at 25°) AEL (Supplemental Material, Figure S2C), and then shifted to 29° till dissection.

For synchronization of larvae, flies were allowed to lay eggs for 3 hr. Newly hatched larvae within a 1-hr interval were collected and transferred onto fresh food plates and aged for specified time periods at 25°.

See also Table S1 for stock details.

### Clonal analyses

To induce zip RNAi clones the following strategy was adopted: the first instar larvae of genotype *hsFLP;ay-GAL4/tub- GAL80^ts^*;*UAS-zip RNAi* were subjected to heat shock for 45 min in a 37° water bath. Post heat shock, larvae were transferred to 18° to suppress Gal4 activity until the early third instar. After that, to express the *zip* RNAi, third instar larvae were reared at 29° till dissection.

To induce ROCK overexpression clones following strategy was adopted: early third instar larvae of genotype *hsFLP*; *ay-GAL4/UAS-ROCK* were subjected to heat shock for 45 min in a 37° water bath and transferred to a temperature of 29° till dissection.

### Immunohistochemistry and imaging

The primary antibodies used in this study include rabbit anti-Ance (1:500, A. D. Shirras; Hurst *et al.* 2003), mouse anti-P1 (1:50, gift from I. Ando), mouse anti-Pxn (1:500, gift from J. Fessler), rabbit anti-proPO (1:2000, gift from M. Kanost), rabbit anti-Hh (1:1000, P. Ingham) mouse anti-Patched (Ptc APA1, 1:30; Developmental Studies Hybridoma Bank), Rat anti-DE-Cadherin (DCAD2, 1:100; Developmental Studies Hybridoma Bank), Rat anti-Ci (1:5, 2A1; Developmental Studies Hybridoma Bank), rabbit pMRLC (1:50; Cell Signaling), and mouse anti-β galactosidase (1:100; Promega, Madison, WI). The following secondary antibodies were used: mouse Cy3, mouse FITC, mouse Dylight 649, rabbit Cy3, rabbit FITC (1:500) (Jackson Immuno-research Laboratories). Lymph glands from synchronized larvae of the desired developmental age were dissected in cold phosphate buffered saline (1× PBS, pH 7.2) and fixed in 4% paraformaldehyde prepared in 1× PBS (pH 7.2) for 45 min (Mandal *et al.* 2007) at room temperature (RT). Tissues were next permeabilized by 0.3% PBT (0.3% triton-X in 1× PBS) for 45 min (3 × 15 min washes) at RT. Blocking was then done in 10% normal goat serum (NGS), for 30–45 min at RT. Tissues were next incubated with the respective primary antibody with appropriate dilution in 10% NGS for 18–20 hr at 4°. Postincubation with the primary antibody, tissues were washed thrice in 0.3% PBT for 15 min each. Next, the tissues were incubated in secondary antibody for 18–20 hr at 4°. The tissues were then subjected to four washes in 0.3% PBT for 15 min each, followed by incubation in DAPI solution (Invitroge, Carlsbad, CA) for 1 hr at RT . Excess DAPI was washed from the tissues using 1× PBS before mounting in Vectashield (Vector Laboratories, Burlingame, CA).

#### Detection of pMRLC expression in lymph gland:

pMRLC staining was performed with a slight modification of the above protocol. Lymph gland from synchronized larvae was dissected in ice-cold PBS and fixed in 4% paraformaldehyde prepared in ice-cold 1× PBS (pH 7.2) for 5 hr at 4°. Tissues were then permeabilized with 0.3% PBS-T (0.3% Tween 20 in 1× PBS). Primary antibody and secondary antibody incubations were performed following the standard protocol.

#### Detection of DE-cadherin expression in lymph gland:

To detect plasma membrane-associated DE-cadherin, the lymph glands were incubated in DE-cadherin antibody (1:50 in PBS) before fixation (Langevin *et al.* 2005) for 1 hr at 4°. Tissues were then fixed in 4% paraformaldehyde prepared in ice-cold 1× PBS (pH 7.2) for 5 hr at 4°. Postincubation primary antibody was washed in 0.3% PBT for 30 min (thrice, 10 min each). Secondary antibody incubation, washes, and mounting were performed following the standard protocol. As a control for the DE-cadherin labeling and *UAS-shg* RNAi construct validation, lymph glands from *TepIV-Gal4/+*; *tub-GAL80^ts^/UAS-shg RNAi* were employed (Figure S5, C’–D’).

#### Detection of extracellular Hh in lymph gland:

Detection of extracellular Hh in the lymph gland was done by the following strategy: synchronized larvae were dissected in cold PBS and fixed in 4% paraformaldehyde prepared in ice-cold 1× PBS (pH 7.2) for 5 hr at 40°. Tissues were washed in 1× PBS for 20 min (twice, 10 min each). Blocking was done in 10% NGS (in 1× PBS) for 30–45 min. Tissues were then incubated with the Hh antibody with appropriate dilution in 10% NGS (1× PBS) for 18–20 hr at 4°. Postprimary-antibody incubation tissues were washed with 0.3% PBT for 45 min (thrice, 15 min each). The standard protocol was followed for the rest of the experiment.

### Phalloidin staining

Dissected samples were fixed in 4% paraformaldehyde prepared in ice-cold 1× PBS (pH 7.2) for 45 min at RT on a shaker. Post fixation, three washes of PBT were done before incubating the tissues in rhodamine-phalloidin (1:100 in PBS) (Molecular Probes, Eugene, OR) for 1 hr. Post incubation, three PBS washes for 10 min each were carried out. Mounting in DAPI Vectashield was done for subsequent imaging.

### EdU incorporation

A Click-iT EdU (5-ethynyl-2’-deoxyuridine, a thymidine analog) kit from Life Technologies was used to perform the DNA replication assay. Lymph glands were dissected in EdU solution (1:1000 in PBS) and kept for 36–40 min at RT for EdU incorporation. The next step was fixation in 4% paraformaldehyde prepared in 1× PBS (pH 7.2) for 45 min at RT. Tissues were then permeabilized in 0.3% PBT (0.3% triton-X in 1× PBS) for 45 min (thrice, 15 min each) at RT. Blocking was then done in 10% normal goat serum (NGS), for 30–45 min at RT. For detecting the incorporated EdU in cells, an azide-based fluorophore was used as described in the manufacturer’s protocol.

### Imaging and statistical analyses

Images were captured using Zeiss LSM 780 and Leica SP5 confocal microscopes. All images were captured as confocal Z sections using the same settings within each set of experiments.

Each experiment was repeated at least thrice to ensure reproducibility. Data are expressed as mean ± SD of values from three sets of independent experiments. Usually, 10 lymph glands were analyzed per genotype and all statistical analyses performed employed a two-tailed Student’s *t*-test. *P* values of <0.05, <0.005, and <0.0005 are considered as statistically significant.

### Quantitative analysis of cell types in lymph gland

For quantifying the percentage of differentiated cells or progenitor cells, three middle stacks from confocal *Z* stack were merged into a single section for each lymph gland using ImageJ/Fiji (NIH) software as previously described (Shim *et al.* 2012). This merged section provides the clearest view of progenitor cells of MZ and the neighboring differentiating cells of the CZ. For images with >1 wavelength, each channel/marker was separately analyzed. Following Shim *et al.*, for estimation of differentiation, P1 positive areas were recalibrated into an identical threshold by using the Binary tool (Process–Binary–Make binary, Image J). The Wand tool automatically captured the area with identical threshold, whereas the size was measured using the Measure tool (Analyze–Measure). To measure the total size of the lobe, recalibration of the DAPI area was done by the Threshold tool until it was overlaid with an identical threshold color. Wand tool was used for selecting this total area for measurement. Percentage of differentiation was estimated by dividing the size of the P1-expressing area by the total size of the lobe (DAPI area). In all cases, per genotype, 10 randomly selected lymph glands were analyzed, and a standard *t*-test was done to evaluate the statistical significance.

#### Quantification of the number of Hh^+^, Ance^+^ and Fucci ^+^ progenitors:

For quantitation of the Hh positive cells in the developing lymph gland, the total number of hhF4-GFP cells in lymph gland was counted using spot detection function in Imaris software (details of the steps involved are illustrated in the figure in supplementary Method S1). Ance-expressing progenitor cells in lymph glands were counted using the surface and spot detection function in Imaris (details of the steps involved are illustrated in the figure in supplementary Method S2). Fucci^+^ progenitors were similarly counted. http://www.bitplane.com/download/manuals/QuickStartTutorials5_7_0.pdf.

#### Quantification of intensity analysis:

To quantify the expression of particular proteins in different genotypes of the lymph gland, we followed the protocol adapted by Shim *et al.* (2012) from http://sciencetechblog.files.wordpress.com/2011/05/measuring-cell-fluorescence-using-imagej.pdf.

**Figure 2:**
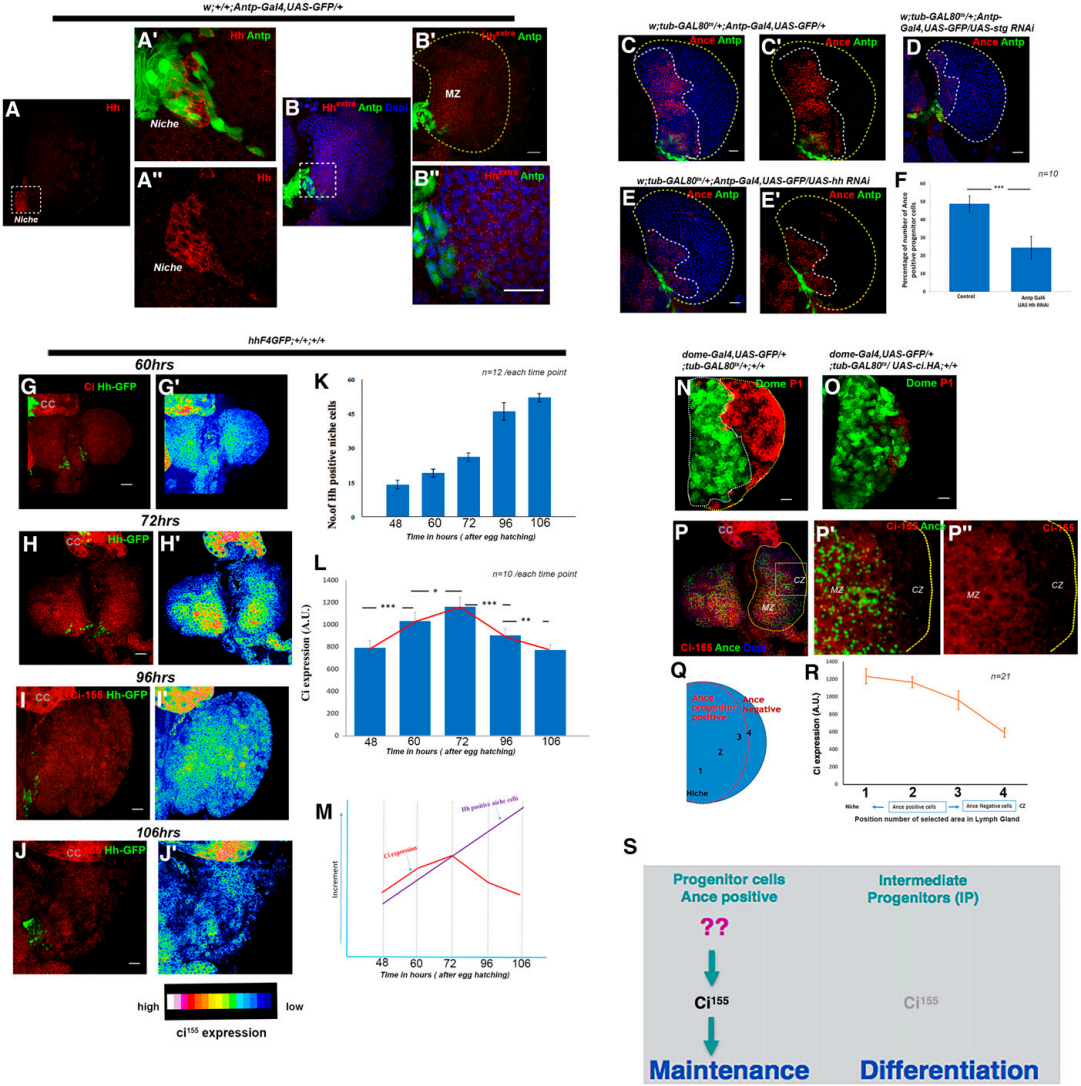
Dynamicity in the expression of Cubitus interruptus in the developing lymph gland. The genotypes are mentioned on the top of the relevant panels. (A–A’’) Morphogen Hh (Antibody, red) expresses in the hematopoietic niche (visualized by *Antp-Gal4*, *UAS-GFP*). (A’ and A’’) Higher magnification of the hematopoietic niche shown in A. (B and B’) Detection of extracellular Hh (red) expression in the progenitors (MZ) by detergent-free permeabilization during antibody labeling. (B’’) Higher magnification of a selected area in B (Antp-GFP marks the niche). (C–F) The level of Hh at the source is critical for progenitor maintenance. Progenitor maintenance declines with the decrease in the number of niche cells by *string* RNAi (Compare C, C’ with D) or Hh is downregulated by *hh* RNAi (compare C with E–F). *P* value for *F* = 2.832 × 10^−8^. Error bars: SD. The timeline of tub-GAL80^ts^ inactivation adopted in all the above experiments is illustrated in Figure S2C. (G–K) As the lymph gland grows, the number of Hh-expressing cells in the niche increases with time. (G–J’) Ci-155 (activated Ci) profiling in 60, 72, 96, and 106 hr AEH. (K) The quantitative analyses of the increment in the number of Hh-expressing niche cells 48, 60, 72, 96, and 106 hr AEH. (L) The quantitative analysis of the Ci intensity throughout development. An increase in Ci expression occurs with the increment in niche number (K) until 72 hr AEH. Despite the increase in the number of Hh-expressing cells post 72 hr AEH (K), a decline in Ci-155 expression is evident in the progenitors (M). *P* value for 60 hr compared to 40 hr = 4.128 × 10^−5^, *P* value for 72 hr compared to 60 hr = 8.521 × 10^−2^, *P* value for 96 hr compared to 72 hr = 4.108 × 10^−7^, and *P* value for 106 hr compared to 96 hr = 6.019 × 10^−3^. (N and O) Compared to control (N) overexpression of activated Ci in the progenitors results in an increase in their number postponing their differentiation (O). (P–P’’) Ci-155 expression (red) is limited to Ance (green)-expressing progenitor cells. A drastic reduction in Ance or Ci-155 occurs in the CZ. (Q and R) Quantitative analyses of Ci-155 level in Ance^+^ (1, 2, and 3) and Ance^–^ (4) progenitor population illustrating the decline in Ci-155 level in Ance^−^ progenitors. The 21 spots analyzed in total are selected based on their distance from Hh-expressing niche and are equidistant from each other. (S) A scheme depicting our hypothesis that a decline in Ci-155 level initiates differentiation. The yellow dotted line marks the whole of the lymph gland in B’, C’, E’, and P–P’’, while the white dotted line marks the progenitors in C’ and E’. The nuclei are marked with DAPI (blue) in B, B’’, C, D, and E. Figures A–F and N–O are of 96 hr AEH. In G–J, the age of the larvae is mentioned in the respective panels. P–P’’ is 72 hr AEH. P and H are identical lymph glands, where three antibodies were analyzed in three different channels: Ci-155 (red), Ance (blue), and GFP to trace Hh expression. For clarity, the Ance expression is represented in green in P. Error Bars = SD. See also Figure S2. Bar, 20 μm.

To quantify Ci intensity in progenitor cells (see Figure 2, G–J’, Figure 4, A–F’, Figure 4, Q–R’, Figure 5, E–F’’, Figure 6, G–H’, and Figure 7, I–K) ImageJ/Fiji software was used to define regions of interest (ROIs) corresponding to progenitor cells, and the mean intensity of each ROI was quantified. For each genotype, at least three ROIs in a lymph gland were defined, and 10 biological samples were quantified.

To analyze Ci intensity profiles in lymph gland with respect to Hh-expressing niche (Figure 2, P–P’’), we measured intensities in four different regions in Ance-positive and Ance-negative progenitors. (Three points equidistant from each other in the Ance-positive domain compared with one in the Ance-negative domain: see Scheme Figure 2.

Sqh-GFP, F-actin, and DE-cadherin intensity analysis:We used ImageJ/Fiji software to analyze the intensity profiles of Sqh:: GFP, F-actin, and DE-cadherin (Shg). For measuring the intensity, a defined line was drawn along the plasma membrane (Cartagena-Rivera *et al.* 2016) in Ance-positive and Ance-negative cells (after background correction) (adapted from Cartagena-Rivera *et al.* 2016; Roubinet *et al.* 2017). For each genotype at least five such measurements were done. Biological replicate was 10.

### Quantitative analysis of *ptc* transcript

The transcript level of *ptc* was analyzed by quantitative RT-PCR (qRT-PCR). RNA was isolated from 150 to 200 whole lymph glands of third instar larvae. In both control and experimental analyses, expression levels of the *ptc* transcript were normalized with respect to *rp49* expression. The control and experiment analyses were repeated thrice, using triplicates each time. The list of primers used is as follows:

*rp49*-forward, 5′-CTAAGCTGTCGCACAAATGGC-3′.
*rp49*-reverse, 3′-TTCTGCATGAGCAGGACCTC-5′.
*ptc*-forward, 5′-TGCACCTCTACGACACCGAA-3′.
*ptc*-reverse, 3′-GGATCTTTACATACGCACCAGC-5′.

### Data and reagent availability

Fly strains are available upon request. The authors affirm that all data necessary for confirming the conclusions of this article are represented fully within the article and its tables and figures. Figures S1–S8, Table S1, and Methods S1 and S2 are available on Figshare. Supplemental material available at FigShare: https://doi.org/10.25386/genetics.8186885.

## Results

### *Drosophila* hematopoietic progenitors are heterogeneous

The expression of two *bona fide* reporters of cell identity can be used to visualize hematopoietic progenitors residing in the lymph gland: *Domeless* (Jung *et al.* 2005) and TepIV (Kroeger *et al.* 2012; Shim *et al.* 2013; Dey *et al.* 2016). Both Domeless (a JAK/STAT receptor; Brown *et al.* 2001; Chen *et al.* 2002) and TepIV (a JAK/STAT-responsive thioester-containing protein four-coding gene; Irving *et al.* 2005) are expressed in second and third instar larvae (Figure 1 and Figure S1, A and A’). However, these progenitors differ in their proliferation status depending on the larval stage (Jung *et al.* 2005). To determine their cell cycle status, we employed the *UAS-FUCCI* system (Figure 1B) (Zielke and Edgar 2015). In this system, a fluorescent ubiquitination-based cell cycle indicator includes two probes. The first probe is an E2F moiety fused to GFP that is degraded during S phase by Cdt2; therefore, cells in G1, G2, and M phase of the cell cycle can be visualized. The CycB moiety fused to monomeric Red Fluorescent Protein (mRFP) acts as the second probe. This moiety is susceptible to degradation by the anaphase-promoting complex/cyclosome (APC/C) during G1 phase and, thus, its presence indicates S, G2, and M phase cells. Hence, cells in G1, S, and G2/early mitosis can be monitored by green, red, and yellow signals, respectively. To further differentiate between G2 and M phase cells, we employed mitosis-specific phospho-histone H3 (PH3) labeling (Jin *et al.* 2015).

Monitoring of the progenitor-specific UAS-FUCCI expression driven by *TepIV-Gal4* demonstrated that the proliferating progenitors were indeed primarily in S phase (red), while a few were in G1 and even fewer were in G2/M (yellow/PH3) (Figure 1C and the quantitative analysis in Figure 1C’). Studies have concluded that these early-stage proliferating progenitors enter quiescence later in development since they do not incorporate EdU (Figure 1D)/BrdU (Jung *et al.* 2005; Mondal *et al.* 2011).

However, analysis of the lymph glands in third instar larvae with the same genotype as above (*TepIV-Gal4*, *UAS-FUCCI*) revealed a G2 arrest (yellow) in the progenitors. Thus, although these cells lack EdU incorporation and PH3 labeling, they have not exited the cell cycle (Figure 1E with a quantification in Figure 1). Therefore, our data demonstrate that the quiescent hemocyte progenitor cells in the lymph gland (Jung *et al.* 2005; Mondal *et al.* 2011) are in G2 arrest.

The above observation prompted us to perform detailed analyses of the nature and state of both second and third instar hemocyte progenitors. In the early second instar lymph gland, where differentiation has not yet started, variable Dome expression was observed. In addition to progenitors with high Dome expression (Dome^high^), some progenitors within an inner core had very low Dome expression (Dome^low^), as assessed via a *dome-Gal4*, *UAS-GFP* construct (Figure 1F). Incidentally, these two subtypes could also be detected by monitoring the expression of Pvf2-LacZ (a transcriptional fusion construct, Figure 1F’; Choi *et al.* 2008), as it corresponds to the Dome^low^ expression (Figure 1, F’’, F’’1, and F’’2). Quite intriguingly, both the Dome^low^ and Dome^high^ progenitors (Figure 1, G and G’) express Ance (red, angiotensin-converting enzyme, Tatei *et al.* 1995), a previously reported marker for third instar hemocyte progenitors (Benmimoun *et al.* 2012) (progenitors visualized by *trol*, the heparin sulfate proteoglycan, Terribly Reduced Optic Lobes, green; Grigorian *et al.* 2013) (Figure S1, B–B’’).

Our temporal analyses revealed that early in development, Ance is present in both the Pvf2^+^ Dome^low^ and Pvf2^−^ Dome^high^ progenitors (Figure S1, C–E’). Later, around the third instar stage, Ance expression overlaps with most of the *dome* expression (Figure 1, H–I1’’) and is mutually exclusive with Peroxidasin expression (Figure 1, J–J1’’), as reported via *Pxn-Gal4*, *UAS*-GFP (Nelson *et al.* 1994). Temporal analyses of *PxnGFP*, Dome, and Ance expression in the same tissue revealed that the progenitors in the peripheral area of the MZ, which co-express Dome and the earliest known differentiation marker Pxn (Figure 1 ) (Sinenko *et al.* 2009), lack Ance expression (Figure S1, F–I’’’). We propose that this group of cells represents the previously identified IP cells (Sinenko *et al.* 2009; Krzemień *et al.* 2010). Interestingly, upon visualization of Pxn by antibody in the lymph glands (*TepIV-Gal4*, *UAS-FUCCI*), it was clear that these cells differ from the rest of the progenitors in their cell cycle status. The majority of the IP cells are cycling (Figure S1, J and J’), while the MZ progenitors are G2 arrested.

At this time point, we observed ongoing expression of Pvf2 (a ligand involved in VEGF signaling; Munier *et al.* 2002) in the inner core of the lymph gland (Figure S1, E and E’).

Based on the above findings, we have classified the *Drosophila* hematopoietic progenitors into three groups: naive progenitors (Pvf2^+^, Ance^+^, Dome^low^), primed progenitors (Pvf2^−^, Ance^+^, Dome^high^), and differentiating IP cells (Ance^−^, Dome^high^, Pxn^+^) (Figure 1, L–L’’). Although previous studies have proposed the presence of IP cells (Sinenko *et al.* 2009; Krzemień *et al.* 2010), the current study provides a marker-based approach for identifying them.

Incidentally, the hemocyte progenitors in the lymph gland depend on Hh signaling for their maintenance (Mandal *et al.* 2007). The niche serves as the source of Hh, and the hematopoietic enhancer of *hh* has been cloned and validated (Tokusumi *et al.* 2010).

Monitoring of Hh expression via antibody staining identified the PSC/niche as the source of Hh (Figure 2, A–A’’). However, upon labeling extracellular Hh, it was further demonstrated that the lymph gland progenitors receive Hh (Figure 2, B–B’’). Based on the functional analyses and expression data (Mandal *et al.* 2007; Tokusumi *et al.* 2010), it appears that Hh signaling originating in the niche is sensed by the MZ progenitors, which then express the downstream transcriptional activator Ci-155 (Figure S2, B and B’), crucial for their maintenance.

Hh signal transduction commences at the plasma membrane where Hh interacts with its 12-transmembrane protein receptor, Patched (Ptc) (Ingham and McMahon 2001). The binding of Ptc and Hh leads to two concurrent and crucial events. First, both Hh and Ptc are internalized by the cells, upon which Hh is targeted to lysosomes for degradation (Gallet and Therond 2005) to limit its spread. Second, once Hh binds to Ptc, the Ptc-mediated repression of the seven-pass transmembrane protein Smoothened is relieved, and Smo is phosphorylated on several residues in a Hh dose-dependent manner (Fan *et al.* 2012). These phosphorylation events lead to Smo activation (Chen and Struhl 1996; Denef *et al.* 2000; Taipale *et al.* 2002; Lum *et al.* 2003), which in turn initiates a signaling cascade to transmit the signal necessary for regulating the Ci transcription factor (Alexandre *et al.* 1996; Méthot and Basler 1999). The outcome of the above events is inhibition of the proteolysis of full-length Ci (Ci-155), which then translocates to the nucleus to activate downstream gene expression (Ingham and McMahon 2001; Lum and Beachy 2004; Lim and Matsui 2010). Interestingly, Ci-155 expression in the mature lymph gland is consistent with the above description. The hemocyte precursor cells near the Hh source (niche) contain abundant Ci-155 (Figure S2A1), while those farther away have lower amounts (Figure S2A2).

Although the role of the Hh released from the niche has been contested by one study (Benmimoun *et al.* 2015), a significant amount of work on the hematopoietic niche has implicated it as either a positive regulator of progenitor maintenance (Mandal *et al.* 2007; Tokusumi *et al.* 2010, 2012, 2015; Mondal *et al.* 2011; Benmimoun *et al.* 2012; Lam *et al.* 2014; Morin-Poulard *et al.* 2016; Grigorian *et al.* 2017; Hao and Jin 2017; Khadilkar *et al.* 2017; Baldeosingh *et al.* 2018; Banerjee *et al.* 2019) or a negative regulator of differentiation (Oyallon *et al.* 2016). To resolve this contradiction, we revisited the role of Hh in this niche. We adopted an approach to determine the role of the niche that differed from the total genetic ablation employed by Benmimoun *et al.* Our argument against overexpressing apoptotic genes in the niche stems from the possibility that inducing apoptosis might have nonspecific effects on the developing lymph gland. Furthermore, many signaling molecules have been identified in this niche, and we believe that total removal of the niche will eliminate all of these signals, some of which might play significant roles in promoting progenitor maintenance or inhibiting differentiation. Furthermore, it has been recently demonstrated that two progenitor subpopulations: Hh-sensitive and Hh-insensitive (Baldeosingh *et al.* 2018; Banerjee *et al.* 2019) exist within the lymph gland. Loss of the niche not only affects the Hh-sensitive population, it also decreases the expression level of the transcription factor Odd in the Hh-insensitive population (Baldeosingh *et al.* 2018).

To circumvent these issues, we used multiple approaches to achieve niche-specific Hh downregulation.

To dampen Hh signaling, we first attempted to reduce the number of Hh-expressing cells in the niche by blocking mitosis. RNAi-mediated downregulation of the cell cycle-regulating gene *string* using two niche-specific GAL4 constructs, *Antp-Gal4*, *UAS-GFP* (Mandal *et al.* 2007) and *pCol-Gal4*, *UAS-2xEGFP* (Krzemień *et al.* 2007; Benmimoun *et al.* 2015), in conjunction with the *GAL80^ts^* allele, was performed following the timeline shown in Figure S2C. This genetic manipulation resulted in a reduction in the number of niche cells (Figure S2, D–H), thereby curtailing the strength of Hh signaling at its source. A drastic decline in the number of progenitors was observed in both cases (Ance labeling, compare Figure 2C with Figure 2D and Figure S2, I and J).

Our second strategy was to downregulate the Hh signaling originating from the niche by directly reducing Hh expression using *UAS-hh RNAi* with the same niche-specific driver lines (*tub-GAL80^ts^*; *Antp-Gal4*, *UAS-GFP/UAS-hh RNAi* and *pCol-Gal4*, *UAS-GFP*; *tub-GAL80^ts^/UAS-hh RNAi*) within a time frame that excluded early proliferation (Figure S2C). The lymph glands in the larvae with these constructs had a substantial decline in progenitor pool compared to the control lymph glands (visualized by Ance: Figure 2, E–F, Figure S2, I–K and Shg: Figure S2, L and M) and an abundance of differentiated cells (P1 marker for the plasmatocytes) (Figure S2, N and O). Likewise, Hh overexpression in the niche resulted in an expansion of the progenitor pool and a reduction in the number of differentiated cells (P1: Figure S2P). These two experiments firmly establish the role of the niche in progenitor maintenance and the requirement of Hh signaling in executing the above process.

Our observations are consistent with other recent findings that affirm the role of Hh signaling originating from the niche as a vital factor in progenitor maintenance (Baldeosingh *et al.* 2018).

Hh expression in the niche is initiated 18 hr after egg hatching (AEH) (Dey *et al.* 2016). However, Ci-155 profiling in the progenitors over the entire larval developmental period revealed an interesting feature. As the lymph gland grew in size, the number of Hh-expressing cells in the niche also increased (visualized by hhF4: a transcriptional reporter of *hh*; Tokusumi *et al.* 2010) (Figure 2, G–J, with a quantitative analysis in Figure 2K). As a result, there was a strengthening of Hh signaling that led to the increased Ci-155 expression in the progenitors until the larvae reach the midthird-instar stage (Figure 2, G’–H’ and quantitative analyses in Figure 2L). Despite a further increase in Hh signaling due to an increase in the number of Hh-expressing cells in the niche after the midthird-instar stage (Figure 2, I–J), we did not observe a further increase in the Ci-155 level in the progenitors (Figure 2, I’–J’ and L). Instead, decreased Ci-155 expression beyond 72 hr AEH was evident from our expression data. The above observation suggested that even though the strength of the Hh signaling increases from its source, there is further developmental regulation on the level of Ci-155 expression in the hemocyte progenitors by the late third instar (Figure 2M) that might be required for their homeostasis. To test this hypothesis, we constitutively expressed an activated form of Ci in the progenitors (*dome-Gal4*, *UAS-GFP*; *UAS-ci.HA/tub-GAL80^ts^*) post hatching. This sustained Ci expression in the progenitors postponed their differentiation and resulted in an increase in their number (Figure 2, N and O). The observation that the cells remain in the progenitor state indicates that regulation on Ci is required for the transition from the progenitor to the differentiated state. Furthermore, an increased number of undifferentiated progenitors were also observed when Smo (a positive regulator of Ci) was overexpressed in the progenitors (Figure S2R). Likewise, Smo downregulation in the progenitors resulted in a reduction in their number (Figure S2Q), reflecting a defect in their maintenance.

Our expression study also revealed a spatial organization of Ci-155 expression that was limited to Ance-expressing progenitors (Figure 2, P–P’’), and that Ci-155 expression was scarcely detectable beyond that domain (IP cells, Figure 2Q). A quantitative analysis of the above data validated our observation that there was, indeed, a sharp decline in the Ci-155 level outside the Ance-positive domain (Figure 2R). Collectively, our results indicate that developmental regulation at the level of the Ci protein is required for progenitor differentiation (Figure 2S).

### Hematopoietic progenitor maintenance requires actomyosin cytoskeleton activity

It has been previously demonstrated that niche-derived Hh and adenosine growth factor A (ADGF-A) produced by differentiated hemocytes regulate the maintenance of *Drosophila* hematopoietic progenitors via Ci-155 upregulation (Mandal *et al.* 2007; Mondal *et al.* 2011; Banerjee *et al.* 2019). Our results clearly demonstrate that constitutive Ci activation in the progenitors supports their maintenance, thereby delaying their differentiation. Therefore, during normal development, the Ci-155 level does not increase beyond the midthird-instar stage to allow progenitor differentiation (despite the time-dependent increase in the number of Hh-expressing cells, compare Figure 2, L and M).

The two known extrinsic signals that maintain the Ci-155 level cannot account for this outcome; therefore, we reasoned that there must be some intrinsic regulation of Hh signaling within the progenitors that helps to maintain their homeostasis.

**Figure 3:**
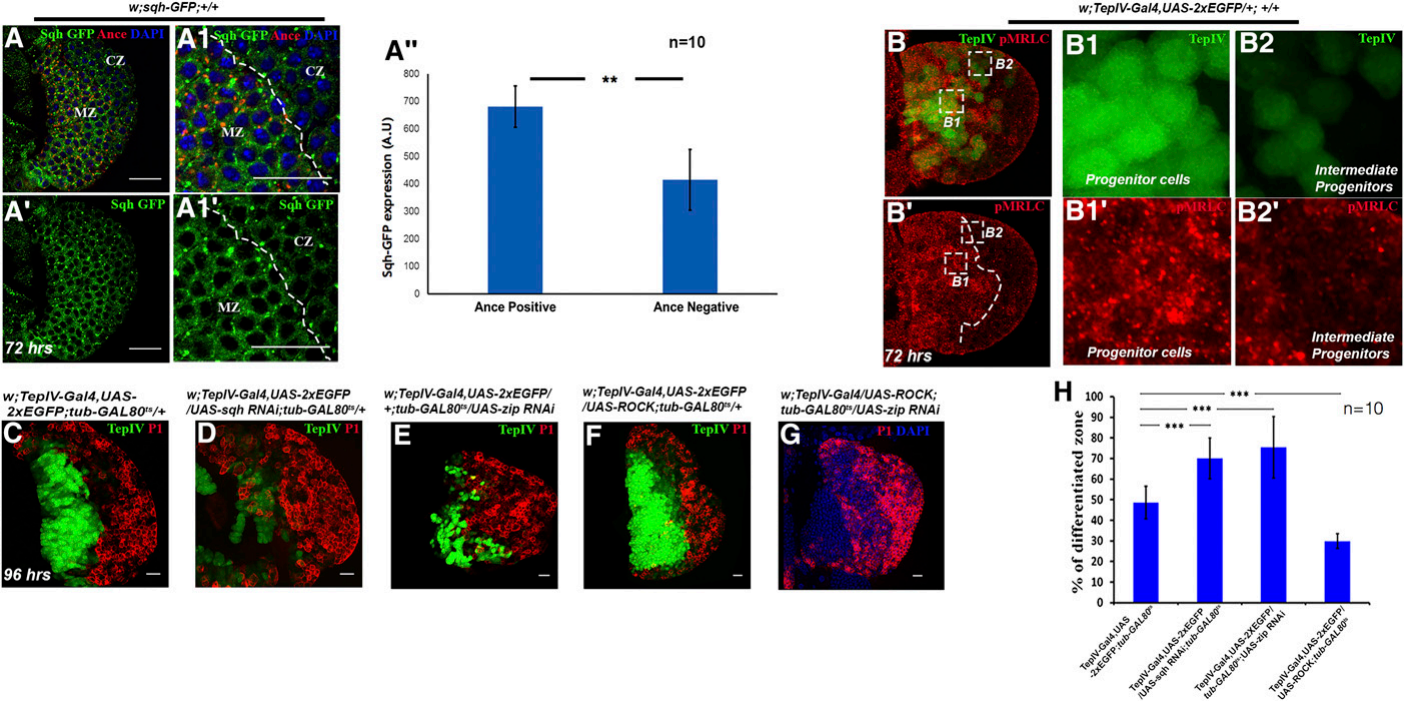
Maintenance of hemocyte progenitors requires activity of the actomyosin network. The genotypes are mentioned on the top of the relevant panels. (A–A1’) Progenitors are enriched in actomyosin components. Ance (red) positive progenitor cells of the third instar lymph gland are enriched in Sqh (green). (A’’) Quantitative analyses of the above result. *P* value: 5.213 × 10^−3^. (B–B2’) pMRLC (red) is also notably enriched in the progenitor population (green, B1), while downregulated in progenitor cells which are in the MZ periphery (IPs, green^low^, B2). (B1 and B2) High magnification of B representing two regions that are distinguishable by high and low *TepIV-Gal4*, *UAS-2xEGFP* (IPs) expression indicating inner and intermediate progenitors (IPs). (C–H) Downregulation of actomyosin components affects progenitors. Compared to Control (C) RNAi-mediated downregulation of Sqh (D) or Zip (E) function affects progenitor number (visualized by *TepIV-Gal4*, *UAS-2xEGFP*). (F) Overexpression of activated ROCK in the progenitors results in an increase in the number of progenitors *(TepIV^+^)*. (G) Co-expression of UAS-*zip* RNAi with activated ROCK fails to sustain the progenitors. (H) Quantitative analyses of the differentiation index in G. *P* value: *TepIV-Gal4, UAS-2xEGFP/UAS-sqhRNAi*; tub-GAL80^ts^ = 1.256 × 10^−6^, *TepIV-Gal4,UAS-2xEGFP; tub-GAL80^ts^/UAS-zipRNAi* = 3.192 × 10^−7^, *TepIV-Gal4,UAS-2xEGFP/UAS-ROCK; tub-GAL80^ts^* = 6.128 × 10^−6^. Error bars = SD. See also Figure S3. Bar, 20 μm.

Recent work on *Drosophila* wing imaginal discs demonstrated that *spaghetti squash* (*sqh*, the regulatory light chain of Myo II) might also be involved in regulating Ci stability (Liu *et al.* 2015). We found that the actomyosin component Sqh (visualized via a *sqhGFP* construct in Figure 3, A–A1’ and quantified in Figure 3A’’) and a marker for activated *sqh*, pMRLC (phosphorylated myosin regulatory light chain; Figure 3, B and B’), were highly enriched in the hemocyte progenitors (Figure 3, B1 and B1’) and downregulated in the lymph gland IP cells (Figure 3, B2 and B2’).

To determine whether a possible *in vivo* genetic link exists between actomyosin function and the elevated Ci-155 level in the hemocyte progenitors, the actomyosin components Sqh and Zipper (*zip*, nonmuscle myosin heavy chain) were individually downregulated via RNAi in the G2-arrested progenitors post 60 hr AEH (following the scheme shown in Figure S2C). Quite strikingly, a decline in the progenitor pool, with a concomitant increase in the number of differentiated cells, was observed in both the cases (Figure 3, C–E and quantified in Figure 3H). Likewise, progenitor-specific overexpression of activated ROCK (Rho-associated protein kinase, ROCK, including its catalytic domain, ROCK) (Amano *et al.* 1996) resulted in an increase in the number of progenitors (Figure 3F). However, ROCK is a multifunctional kinase involved in cell behavior, motility, and centrosome positioning via collaborations with diverse signaling pathways (Riento and Ridley 2003; Schlessinger *et al.* 2009; Bryan *et al.* 2010; Noma *et al.* 2012). To evaluate whether the effect seen on overexpressing activated ROCK was indeed via the actomyosin network, we downregulated Zip in the progenitors in which ROCK was constitutively activated. While ROCK overexpression alone resulted in a significantly lower percentage of differentiation compared to that in the control cells, loss of *zip* in the same background rescued this phenotype (compare Figure 3F with Figure 3G and Figure S3A).

These experiments suggest that actomyosin activity regulates progenitor maintenance akin to the mammalian system in which actomyosin-derived biophysical force is required for hematopoietic stem cell and progenitor maintenance (Adamo *et al.* 2009; Shin *et al.* 2014; Choi and Harley 2017).

### Actomyosin network regulates progenitor maintenance via Ci activity

**Figure 4:**
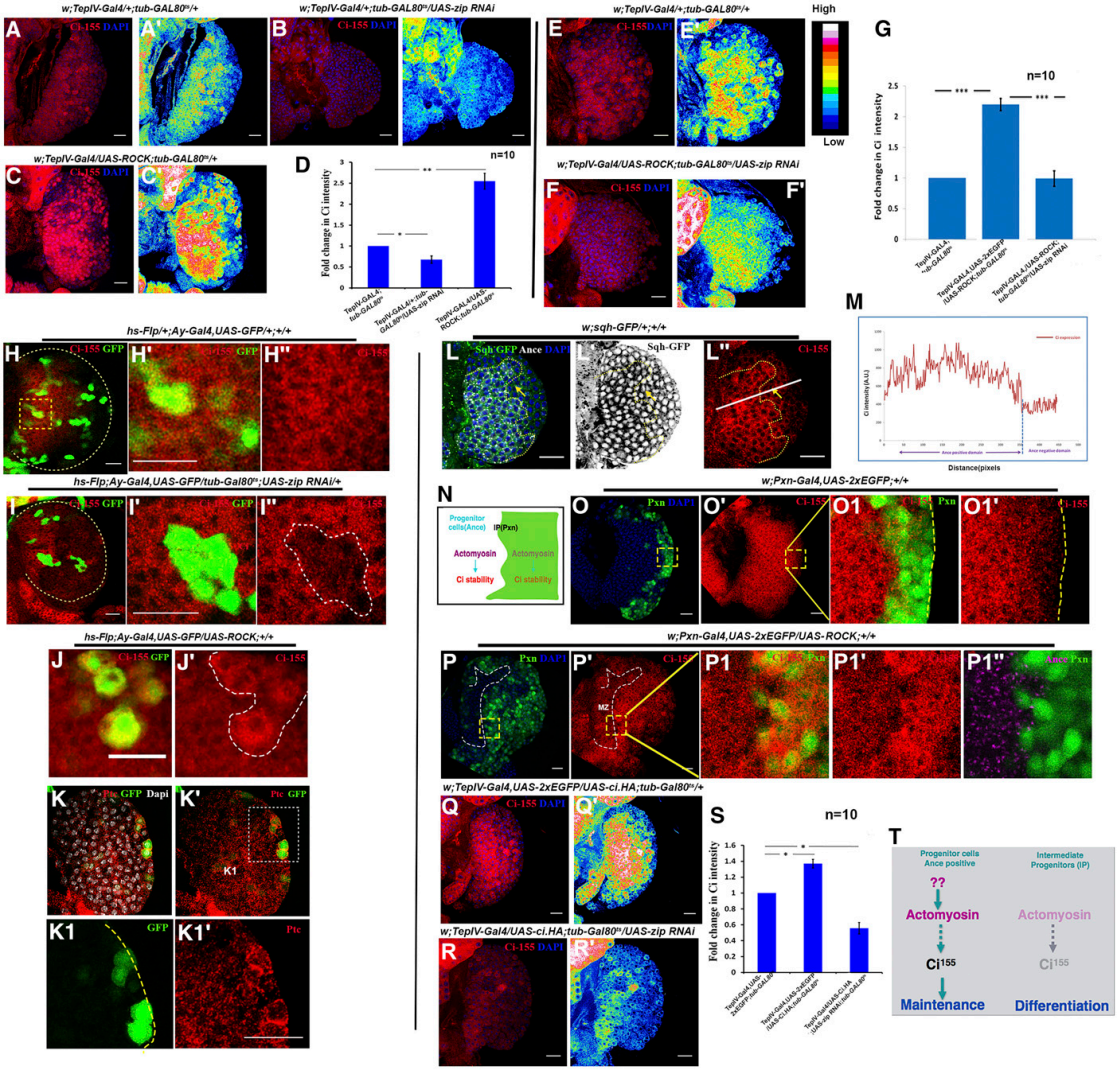
The actomyosin network regulates progenitor maintenance through Ci activity. The genotypes are mentioned on the top of the relevant panels. (A–D) RNAi-mediated downregulation of Zip (B and B’) results in lowering of the Ci-155 level, while overexpression of ROCK causes an increment of Ci-155 in the progenitors (C and C’). (D) Quantitative analyses of the above result. *P* value: *TepIV-Gal4*; *tub-GAL80^ts^/UAS-zipRNAi* = 2.146 × 10^−3^ and *TepIV-Gal/UAS-ROCK*; *tub-^ts^* = 4.97 × 10^−3^. (E–G) Co-expression of *zipRNAi* with activated ROCK in progenitors fails to sustain the Ci-155 expression (F and F’) compared to control (E and E’) leading to the decline in progenitor number as seen in Figure 3G. (G) Quantitative analyses of Ci-155 in the above experiment. *P* value for TepIV-Gal4, UAS-2xEGFP/UAS-ROCK; tub-GAL80^ts^ = 4.614 × 10^−3^ compared to control and *P* value for TepIV-Gal4/UAS-ROCK; tub-GAL80^ts^/UAS-zipRNAi = 2.63 × 10^−4^ compared to TepIV-Gal4, UAS-2xEGFP/UAS-ROCK; tub-GAL80^ts^ (H–I’’) Ci-155 expression decreases in *hsFLP;ay-GAL4/tub-GAL80^ts^*; *UAS-zip RNAi* clones (green) (I–I’’) compared to mock clones (H–H’’) and neighboring cells. (J–K2’) *hsFLP*; *ay-GAL4/UAS-ROCK* clones (green) have upregulated Ci-155 (red: J and J’) as well as upregulated Ptc expression (red: K–K1’) compared to the neighboring wild-type cells. (K1 and K1’ are the higher magnification of K denoted by the box in K’.) (L–L’’) Co-labeling of Sqh GFP, Ance, and Ci-155 antibody, a drastic decline in the Ci-155 level in the cells next door to the Ance-expressing progenitors is evident (arrow, IP). (M) The intensity profile of Ci-155 in Ance-positive and Ance-negative progenitors (along the line drawn in L’’) reflects a stark decline in the level in Ance- progenitors. (N) A scheme based on our hypothesis. (O–P’) Sustained activation of *sqh* in IP stabilizes Ci-155 (P–P1’) compared to control (O–O1’). (P1’’) This sustained Ci-155 expression in IP cells does not revert them to the progenitor state (evidenced by lack of Ance: purple). (O1 and O1’) Higher magnification of an ROI selected from O’ and P1–P1’ are the higher magnification of regions selected from P’. (Q–S) Overexpression of activated Ci is unable to maintain the Ci-155 level in zip loss from progenitors compared to control. (S) Quantitative analysis of Ci expression. *P* value: TepIV-Gal4, UAS-2xEGFP/UAS-ci.HA, tub-GAL80^ts^ = 5.512 × 10^−2^. TepIV-Gal4/UAS-ci.HA; tub-GAL80^ts^/UAS-zipRNAi = 7.371 × 10^−3^. (T) A scheme based on our findings. In all cases except clones where tub-GAL80^ts^ has been used in conjunction with TepIV-Gal4, the strategy followed for Gal4 activation is represented in Figure S2C. Clonal induction timeline is shown in Figure S4, C and D. Figures H–J’ are of late third instar. The rest of the lymph glands are midthird instar. *UAS-2xGFP* construct was included in *Tep-Gal4IV*, *UAS-ci.HA*, *tub-GAL80^ts^* to serve as a proper control for the two UAS construct used in Q: *Tep-Gal4IV/UAS-ci.HA*; *tub-GAL80^ts^/UAS-zipRNAi*. The nuclei are marked with DAPI (blue) in A–C, E, F, Q, and R. Error bars = SD. See also Figure S4. Bar, 20 μm.

Interestingly, there was a decrease in the level of the maintenance factor Ci-155 upon Zip or Sqh downregulation compared to that of the control (Figure 4, A–B’ and D and Figure S4, A and B’), while a twofold increase in Ci-155 abundance was observed upon ROCK overexpression (Figure 4, C, C’, and D). In the ROCK-activated progenitors restoration of this drastically elevated level of Ci-155 expression to the wild-type level (Figure 4, E and E’) was achieved via Zip downregulation (compare Figure 4, F and F’ with Figure 4, C and C’ and Figure 4G). The recovery of the Ci-155 level, in turn, led to a rescue of the arrested differentiation observed in ROCK-overexpressing progenitors (compare Figure 3F with Figure 3G and Figure S3A).

Collectively, the above results indicate that actomyosin network positively regulates hemocyte progenitor maintenance via intrinsic Ci-155 regulation.

To determine whether activity of actomyosin component regulates Ci-155 expression cell-autonomously in the lymph gland, *zip RNAi* clones were generated using Ay-GAL4 (see Figure S4C). No change in Ci-155 expression was observed in lymph glands with the mock clones (Figure 4, H–H’’); however, the lymph glands with the *zip RNAi* clones (marked by GFP) exhibited decreased Ci-155 levels compared to that in the neighboring wild-type cells (Figure 4, I–I’’ and Figure S4, E and E’).

Quantitative analyses revealed that 60% (7 out of 12) of the *zip RNAi* clones analyzed had a reduction of 60% in the level of Ci-155 expression (Figure S4F).

Furthermore, upregulated Ci-155 expression was observed in lymph gland clones that overexpressed ROCK compared to that in the neighboring wild-type cells (Figure 4, J and J’ and Figure S4D). Similarly, increased Patched expression was also observed in the ROCK-overexpressing clones (Figure 4, K–K1’ and Figure S4, G–G’’), attesting to the fact that higher Ci-155 expression is sufficient to trigger the expression of downstream Hh-target genes (Chen and Struhl 1996). Patched activation was also detected when the *ptc* transcript level was analyzed upon progenitor-specific ROCK overexpression (*TepIV-Gal4/UAS-ROCK*; *tub-GAL80^ts^/+*, Figure S4H).

Taken together, these results demonstrate that the actomyosin network cell-autonomously regulates the Ci-155 level in lymph gland progenitors.

Co-labeling of Ance, Sqh, and Ci-155 revealed that the Ci-155 level decreased drastically in IP cells (IP, Sqh^low^ and Ance^–^ in gray) compared to its expression level in the Ance-expressing progenitors in the MZ (Figure 4, L–L’’, Sqh^high^). A quantitative analysis of the Ci-155 intensity profile (indicated by the line in Figure 4L’’) validated the above finding (Figure 4M). This robust Ci-155 downregulation in the IP cells suggests altered Ci stability, which would allow progenitor differentiation (Figure 4N). Therefore, we supposed that if the decrease in expression of cortical actomyosin in the IP cells explains the low Ci-155 expression level, then sustained *sqh* activation via ROCK in this population should maintain higher Ci-155 expression. Indeed, upon ROCK overexpression, high Ci-155 levels were observed in the IP population carrying the *Pxn-Gal4*, *UAS-GFP/UAS-ROCK* construct (Figure 4, P–P1’’) compared to the low levels observed in the control IP cells (Figure 4, O–O1’). However, as shown by the lack of Ance labeling, the sustained Ci-155 level in the above genetic background failed to revert the IP cells back to the progenitor state (Figure 4P1’’). It is also clear from this experiment that sustained Ci activity in the differentiated and intermediate progenitors can lead to increased proliferation (EdU incorporation, Figure S4, I–J’ and K–L’) that disturbs normal lymph gland patterning without affecting cell-fate specification assayed by markers for both plasmatocyte (P1) and crystal cell (ProPO) (Figure S4, M–O).

Canonical Hh signaling regulates the Ci-155 level by inhibiting the PKA protein kinase. When Hh is available, PKA phosphorylates and activates Smo (Chen and Jiang 2013; Li *et al.* 2014). This upregulation of Smo activity blocks PKA-dependent proteolytic processing of Ci-155, thus preventing formation of the repressor Ci-75 (Chen and Jiang 2013; Ranieri *et al.* 2014). We wondered whether regulation of the Ci level in this context also depends on canonical PKA-mediated regulation. To investigate this possibility, we co-expressed *UAS-zipRNAi* and a UAS Ci variant (ci.HA) that lacks PKA phosphorylation sites, thus rendering it resistant to PKA-mediated degradation (Chen *et al*. 1999). Interestingly, under these conditions, the progenitors could not maintain Ci-155 expression, despite constitutive expression of activated Ci (*TepIV-Gal4/UAS-ci.HA*; *UAS-zip RNAi/+*, compare Figure 4, Q and Q’ with Figure 4, R and R’ and with a quantification in Figure 4S).

These results demonstrate that activity of the actomyosin network regulates the Ci-155 expression in hemocyte progenitors that is essential for their maintenance (Figure 4T). These data also show that this modulation is independent of the PKA activation responsible for fine-tuning the Ci activity (Mondal *et al.* 2011) to maintain progenitor homeostasis.

### Cell–cell adhesion mediates activity of the actomyosin components in hemocyte progenitors

**Figure 5:**
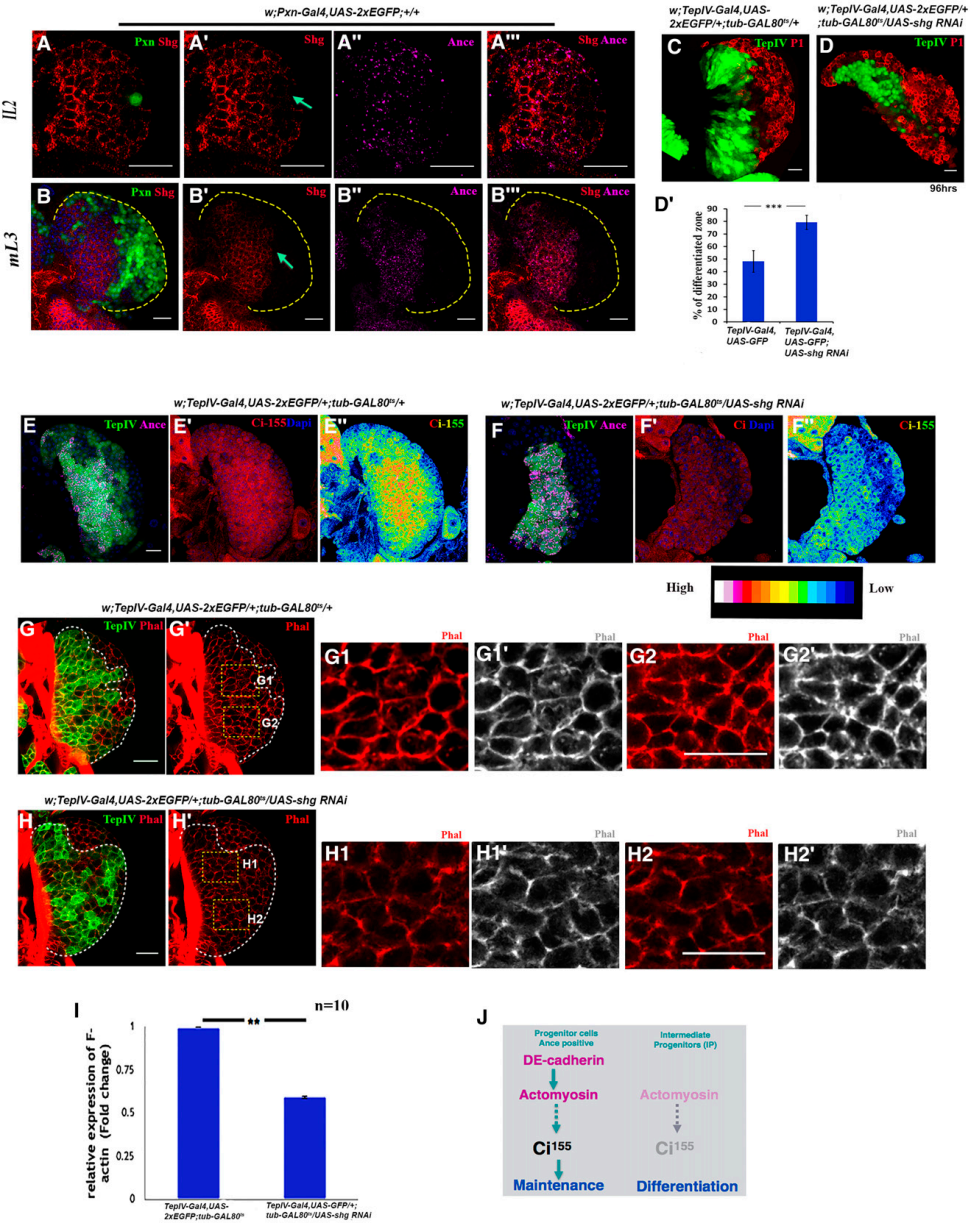
DE-cadherin mediates the activity of actomyosin network within the hemocyte progenitors. The genotypes are mentioned on the top of the relevant panels. (A–B’’) Ance-positive progenitor cells (magenta) are highly rich in DE-cadherin. Throughout development, DE-cadherin expression (arrow in A’ and B’) is downregulated in the intermediate progenitor (visualized by Pxn GFP). In A–A’’ the age of the larvae is late second instar (lL2) whereas B–B’’ it is midthird instar (mL3). (C and D) Downregulation of Shg function in the progenitors affects their numbers in *TepIV-Gal4*, *UAS-2xEGFP*; *tub-GAL80^ts^/UAS-shg RNAi* compared to control. Progenitors visualized by *TepIV-Gal4*, *UAS-2xEGFP* (green) and differentiated cells by P1 (red). (D’) Quantitative analyses of the above result reveal 1.5-fold increment in differentiation index. *P* value = 3.126 × 10^−7^. (E–F’’) Compared to Control (E–E’’) Ci-155 expression is drastically affected in the progenitors upon loss of Shg (F–F’’). Progenitors are visualized by *TepIV-Gal4*, *UAS-2xEGFP* (green) and Ci-155 in red. (G–H2’) F-actin assembly in progenitors declines upon downregulation of DE-cadherin (Shg) when compared to control (compare G–G2’ with H–H2’). Progenitors are marked by *TepIV-Gal4*, *UAS-2xEGFP* (green) and Phal (red and gray). G1 and G2 are areas selected for higher magnification (G’), while H1 and H2 are the higher magnification of areas selected from H’. (I) Quantitative analyses of the above results show a noticeable decline in (F) actin expression level. *P* value = 8.319 × 10^−3^. (J) A scheme based on our above finding linking DE-cadherin genetically to Ci-155 expression via actomyosin assembly and bringing about progenitor maintenance in MZ, whereas downregulation of the entire network facilitates progenitor differentiation. Figures C–D’ are of 96 hr AEH. The rest of the figures are of lymph glands from 72 hr AEH. The nuclei are marked with DAPI (blue) in B, E, E’, F, and F’. Error bars = SD. See also Figure S5. Bar, 20 μm.

Next, we addressed how cortical actomyosin enrichment occurs specifically in the progenitors (Figure 4T). Studies have revealed that several factors control actomyosin assembly, including DE-cadherin-mediated cell–cell adhesion (Millán *et al.* 2010; Cai *et al.* 2014; Hunter *et al.* 2015; Jurado *et al.* 2016). Moreover, cortical tension and adhesion are related processes, as both depend on E-cadherin complexes (Millán *et al.* 2010). Intriguingly, hemocyte progenitors in the lymph gland also express DE-cadherin (Jung *et al.* 2005), which has previously been linked to progenitor maintenance (Gao *et al.* 2013); however, the underlying mechanism of its role is not known. This missing information encouraged us to analyze the spatial and temporal expression of DE-cadherin/Shotgun (Shg) in relation to Ance expression in the progenitors in which the differentiating cells are marked with *PxnGFP*. Co-expression studies revealed DE-cadherin/Shg enrichment in the Ance-positive progenitors (Figure 5, A–B’’’ with the quantification in Figure S5B). It is also clear that Shg is highly enriched in the inner core of the *domeGFP*-expressing progenitor cell population, while it is downregulated in the peripheral *domeGFP*-expressing progenitors (indicated by the arrow in Figure S5, A and A’), an expression pattern that might be necessary for their differentiation.

Interestingly, upon Shg downregulation in the progenitors (performed in the *TepIV-Gal4*; *tub-GAL80^ts^/UAS-shg RNAi*, Figure S5, C–D’) following the same timeline used for the actomyosin studies (Figure S2C), both the number of progenitors (Figure 5, C–D’) and Ci-155 expression declined (Figure 5, E–F’’). These phenotypes are reminiscent of those resulting from loss of actomyosin components (Figure 3, C–E and Figure 4, A–B’). Quantification revealed a 1.5-fold increase in the differentiation index (Figure 5D’) in the above genetic background. Various studies in *Drosophila*, as well as in mammalian systems, have revealed that cadherins cooperate with the actin-binding protein α-catenin and that they are involved in tethering the actin cytoskeleton to the plasma membrane (Abe and Takeichi 2008; Buckley *et al.* 2014; Chen *et al.* 2015; Nelson and Weis 2016). The tethering of the actin cytoskeleton to the plasma membrane leads to recruitment of nonmuscle myosin II, which then forms a mechanically active actomyosin network (Kasza and Zallen 2011; Jodoin *et al.* 2015). Thus, we next asked whether loss of DE-cadherin/Shg in the progenitors would alter F-actin expression.

Upon Shg downregulation, F-actin expression drastically decreased in the progenitors (Figure 5, G–H’). Higher magnification images revealed that Shg downregulation indeed disturbed the F-actin organization near the plasma membrane (Figure 5, G1–G2’ and H1–H2’).

An analysis of the cortical F-actin level (assayed by drawing a line along the plasma membranes of progenitors; Cartagena-Rivera *et al.* 2016) revealed that the DE-cadherin downregulation in the hemocyte progenitors positively affected the level of cortical F-actin (Figure 5I, Phal).

These observations indicated that both DE-cadherin and the actomyosin network regulate the Ci-155 level required for progenitor maintenance (Figure 5J).

### Ance is a genetic modifier of cell–cell adhesion and the actomyosin network that regulates Ci stability

In addition to actomyosin components and DE-cadherin, the hemocyte progenitors also express *Ance* throughout their lifetime. ACE (angiotensin-converting enzyme) is also present in vertebrate blood stem cells and - progenitor cells where it functions as a component of the renin–angiotensin–aldosterone system (RAAS), a dedicated signaling pathway responsible for regulating blood pressure (Bernstein *et al.* 2012). However, it has been proposed that ACE also functions outside of its complex collaboration with the RAAS (Kohlstedt *et al.* 2006; Lambert *et al.* 2010). Previous studies in vertebrates have teased out a requirement for ACE in blood cell development, along with the other RAAS components (Rousseau-Plasse *et al.* 1998; Jokubaitis *et al.* 2008; Sinka *et al.* 2012). Thus, a blood-specific role of ACE in hematopoiesis, in addition to its role in the RAAS, has not yet been elucidated. Since *Drosophila* encodes Ance despite having no RAAS, we speculated that flies might be an excellent system to gain insight into a blood-specific role for ACE.

**Figure 6:**
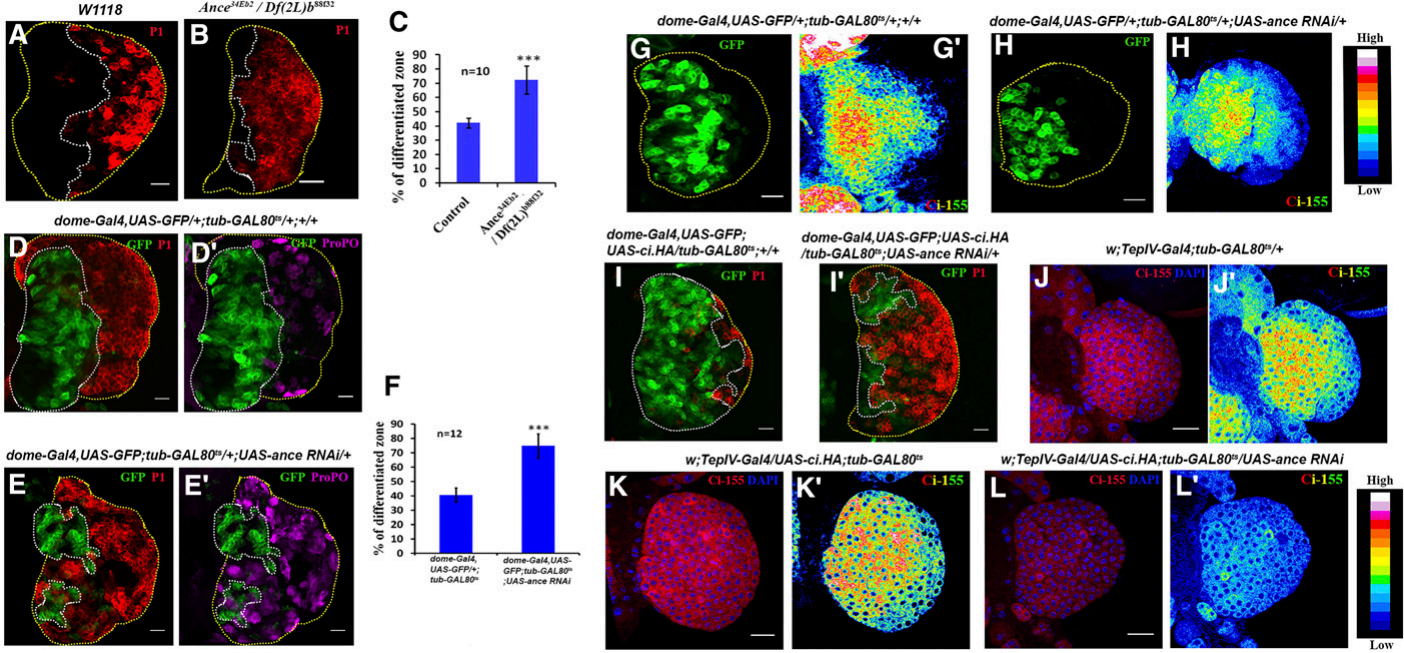
Ance is a genetic modifier of the cell–cell adhesion and actomyosin network that regulates Ci activity. The genotypes are mentioned on the top of the relevant panels. (A–C) Hetero-allelic combination of Ance^34Eb2^/Ance deficiency. (B) shows a significant increase in differentiation when compared to the wild-type (A). (C) Quantitative analysis of differentiating cells in wild type and *Ance^34Eb2^/Ance def* (*P* = 1.58 × 10^−6^). (D–F) RNAi-mediated downregulation of Ance (E) using *dome-Gal4*, *UAS-GFP*; *tub-GAL80^ts^*; *UAS-ance RNAi* in G2 arrested progenitor leads to increase in plasmatocytes (in red) compared with control (D). Crystal cells also increase upon Ance downregulation (E’ compare with D’, control). (F) Quantitative analysis of differentiating cells in control and upon Ance downregulation by RNAi (*P* value = 4.22 × 10^−10^). (G–H’) Upon Ance downregulation from the progenitors, the Ci-155 expression is significantly reduced even at 72 hr AEH compared to control. Please note: Since at 96 hr the entire lymph gland is differentiated upon Ance loss, we have analyzed at 72 hr AEH where few progenitors can still be detected. (I and I’) Compared to control, overexpression of ci.HA in the progenitor (I) posthatching led to their increment in number (*dome-Gal4*, *UAS-GFP*). I’ shows that simultaneous removal of Ance function overrides ci.HA activity. (J–L’) ci.HA overexpression in progenitors results in increase of Ci-155 domain (K and K’) when compared to control (J and J’). However, when Ance is downregulated in the progenitors of the above genotype, they are unable to sustain high Ci-155 level (L and L’). Figures A–F and I and I’ are of 96 hr AEH. The rest of the lymph glands are from larvae of 72 hr AEH. The nuclei are marked with DAPI (blue) in J, K, and L. Error bars = SD. See also Figure S6. Bar, 20 μm.

Studies in mammalian endothelial cells (Kohlstedt *et al.* 2004, 2006) have revealed a strong association between ACE and actomyosin (MYH9: myosin heavy chain). In this context, angiotensin is essential for MYH9 activation; therefore, we hypothesized that Ance might genetically interact with actomyosin components and that this interaction might be important for progenitor maintenance. An *Ance* mutant strain (heteroallele: *Ance^34Eb2^*/Df (2L)b^88f32^; Hurst *et al.* 2003) exhibited a drastic decrease in the number of progenitors and a concomitant increase in the number of differentiated cells (based on the P1 marker for plasmatocytes; Figure 6, A and B) compared with the cell numbers in control lymph glands of the same age. Quantification of the above results revealed a 1.5-fold increase in the differentiation index (Figure 6C). To confirm the above observation, RNAi-mediated downregulation of *Ance* function was performed in the same time frame (Figure S2C) used in the experiments in which Shg and actomyosin components were downregulated in G2-arrested progenitors (using either *dome-Gal4* or *TepIV-Gal4*, respectively). Ance downregulation resulted in a sharp decline in the number of progenitors (visualized by *GFP*) and increased differentiation (P1: red and proPO: magenta, marking the crystal cells), mimicking the phenotypes associated with loss of Shg or actomyosin network (Figure 6, D–E’). Quantification of the above results revealed a 1.5-fold increase in the differentiation index (Figure 6F). We next assayed the Ci-155 expression in progenitors with impaired *Ance* function. For this analysis, we examined cells 72 hr AEH, as Dome*GFP* expression is completely absent by 96 hr AEH due to the ectopic differentiation that occurs in the absence of Ance. At this time point (72 hr AEH), progenitors could still be seen with ectopic differentiation (Figure S6, A and B). Our data showed that the Ci-155 level was significantly reduced upon Ance downregulation (Figure 6, H and H’) compared to the level in control lymph glands of the same age (Figure 6, G and G’). We further wondered whether overexpressing ci.HA in progenitors carrying the *TepIV-Gal4*; *tub-GAL80^ts^/UAS-ance RNAi* construct might rescue this phenotype. There was a robust increase in the number of progenitors and domain of Ci-155 expression upon ci.HA overexpression (Figure 6I and compare Figure 6, J and J’ with Figure 6, K and K’). However, in the lymph glands expressing the *TepIV-Gal4/UAS-ci.HA*; *tub-GAL80^ts^/UAS-ance RNAi* construct, a drastic reduction in progenitor number with a corresponding decline in Ci-155 expression (Figure 6, L and L’), accompanied by an increase in differentiation, was observed (Figure 6I’).

Mirroring the effects of *shg* and actomyosin network downregulation, the number of progenitors was also noticeably reduced (Shg: Figure 5, E–F’’ and actomyosin: Figure 3, C–E and Figure 4, A–B’).

Our expression studies, along with our genetic analyses, indicate that Ance and the Shg-actomyosin network might interact to ensure hemocyte progenitor homeostasis.

**Figure 7:**
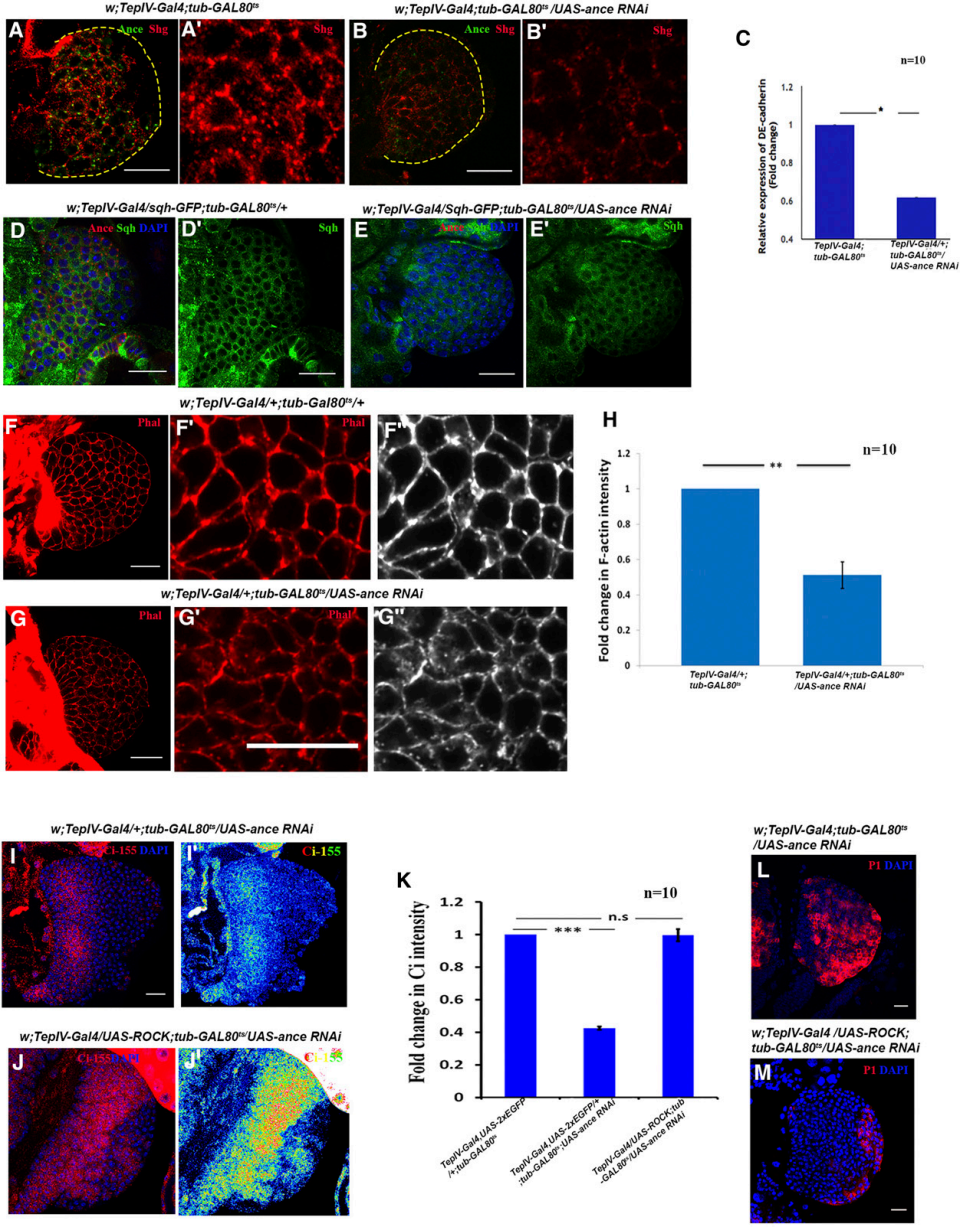
DE-cadherin and the expression of actomyosin components declines upon downregulation of Ance. The genotypes are mentioned on the top of the relevant panels. (A–B’) Shg expression (red) drastically declines in the plasma membrane of the progenitors (*TepIV-Gal4;tub-GAL80^ts^/UAS-anceRNAi*) compared to control. (C) Quantitative analyses of the above result validate the downregulation of Shg upon loss of Ance function. *P* value = 9.43 × 10^−3^. (D–G’’) Reduction in sqh-GFP expression (D–E’) and cortical F-actin level (F–G’’) is evident upon Ance downregulation from the progenitor cells. (H) Quantitative analyses of the F-actin level upon Ance loss, *P* value = 7.781 × 10^−3^. (I and I’) Loss of Ance from progenitors affects Ci-155 level. However, expression of UAS-ROCK in this genotype can rescue the Ci-155 expression (J and J’) and thereby the maintenance defect seen upon loss of Ance (L and M). (K) Quantitative analyses of the above result; *P* value for TepIV-Gal4, UAS-2xGFP;tub-GAL80^ts^/UAS-anceRNAi = 6.35 × 10^−5^ *P* value for TepIV-Gal4/UAS-ROCK; tub-GAL80^ts^/UAS-anceRNAi is 9.003 × 10^−1^. Figures L and M are of 96 hr AEH. The rest of the lymph glands are from larvae of 72 hr AEH. The nuclei are marked with DAPI (blue) in D, E, I, J, L, and M. A UAS-2xEGFP construct was included in (I) TepIV-Gal4, UAS-2xEGFP;tub-GAL80^ts^/UAS-anceRNAi to serve as a proper control for the two UAS constructs used in J: TepIV-Gal4/UAS-ROCK; tub-GAL80^ts^/UAS-anceRNAi. Error bars = SD. See also Figure S7. Bar, 20 μm.

We next asked where Ance fits into the proposed network. Accumulation of the cell adhesion molecule DE-cadherin/Shg on the plasma membrane of the progenitors was drastically reduced upon loss of Ance (compare Figure 7, A–B’ and Figure 7C). Quite intriguingly, monitoring the sqh-GFP expression after *Ance* knockdown revealed a reduction in GFP expression in the progenitors (Figure 7, D–E’ with a quantitative estimation in Figure S7A). A decline in the cortical F-actin level was also observed upon Ance downregulation in the hemocyte progenitors (Figure 7, F–G’’ and quantified in Figure 7H). The above results suggest a strong link between Ance function and increase in the activity of the actomyosin network in hemocyte progenitors, reflecting a similar relationship to that reported in endothelial cells (Kohlstedt *et al.* 2006). To further investigate the sqh phosphorylation status in progenitors with impaired Ance function, the pMRLC antibody was used. In contrast to wild-type progenitors, in which the pMRLC expression was enriched similarly to the sqh-GFP expression level, a reduction in the level of pMRLC expression was observed upon RNAi-mediated downregulation of Ance function (Figure S7, B–C’’).

Remarkably, ROCK overexpression rescued the Ci-155 expression in progenitors lacking Ance (Figure 7, I–J’). A quantitative analysis of the intensity profile of the Ci-155 level in the above experiment confirmed that it was indeed comparable to that in the control (Figure 7K). As a consequence of this effect, the maintenance defect caused by loss of Ance was also rescued (Figure 7, L and M). Based on these results along with our findings that downregulation of Shg and actomyosin components in progenitors resulted in phenotypes identical to those resulting from loss of Ance, we reasoned that Ance, Shg, and components of actomyosin genetically interact to ensure hemocyte progenitor maintenance. Thus, we propose that Ance, Shg, and the actomyosin network regulate Ci activity to positively modulate Hh-mediated signal transduction.

### Hh signaling does not regulate cell–cell adhesion or actomyosin assembly in hematopoietic progenitors

Quite intriguingly, Hh has been linked to the regulation of cell–cell adhesion and actomyosin contractility (Jarov *et al.* 2003; Fernandes *et al.* 2014; Lai *et al.* 2017). We wondered whether in lymph glands, in addition to modulating Ci activity via PKA, Hh might fine-tune the activity of the Ci protein via a second axis involving cell adhesion and the actomyosin network. Although the number of hemocyte progenitors was much lower upon loss of Hh from the niche (Figure S8, B and B’) or Smo from the progenitors (Figure S8, C and C’), the Shg expression level in the residual progenitors was comparable to that in the control cells (Figure S8, A, A’, and E). Overexpression of the Hh signaling component Smo in the progenitors led to an increase in their number, while the Shg expression level was similar to that in the wild-type cells (Figure S8, D, D’, and E). Thus, neither loss nor gain of Hh signaling affects cell–cell adhesion in the progenitors, as indicated by the absence of changes in Shg expression. Actomyosin assembly (as assayed by sqh-GFP expression) upon loss of Hh from the niche was also comparable to that in the wild-type cells (Figure S8, F–G’, and H). From these results, we conclude that Hh signaling does not regulate cell–cell adhesion or actomyosin assembly in hematopoietic progenitors.

## Discussion

The tightly packed progenitors in the core of the lymph gland are enriched in DE-cadherin, while those at the periphery tend to lose it. The loss of DE-cadherin allows these cells to increase their spacing and granularity to initiate the differentiation program (Banerjee *et al.* 2019). Our study reveals that DE-cadherin function is linked to actomyosin activity in hemocyte progenitors since its downregulation alone affects actomyosin assembly and, consequently, progenitor maintenance. In this context, cell–cell adhesion and the actomyosin network cooperatively function as a sensor of progenitor identity to maintain progenitor cell homeostasis.

Although the hematopoietic stem cells in first instar larvae are in S phase (Dey *et al.* 2016), our study shows that the late larval lymph gland progenitors in *Drosophila* are in the G2 phase of the cell cycle. These blood progenitors initially undergo proliferation to create a progenitor pool in which the cells ultimately arrest in G2 phase. We believe that as far as the hemocyte progenitors are concerned, exiting the cell cycle offers no benefit. In fact, the G2 arrest actually maintains a primed progenitor population ready to respond to developmental cues or immune challenges. This G2 arrest provides a unique opportunity to uncouple the signals relevant for maintenance *vs.* those involved in differentiation, independent of influence from the cell cycle. It is important to note that G2 arrests are often part of normal development of the germ line (Morris and Spradling 2011), intestinal stem cells (Zielke *et al.* 2016), neuronal stem cells (Otsuki and Brand 2018), and sensory organ precursors of *Drosophila* (Meserve and Duronio 2017), the primordial germ cells in *C*. *elegans* (Seidel and Kimble 2015), and the tailbud progenitors in zebrafish (Bouldin *et al.* 2014). These multiple instances of G2 arrest suggest that it has been preferably adopted by diverse progenitors. In the case of the hematopoietic progenitors, it might also be possible that the G2 arrest is linked to increased responses to particular signaling pathways that regulate lineage commitment or increase maintenance efficiency.

The population of hematopoietic progenitors that express Domeless and Tep IV was thought to be homogeneous. Although various studies have proposed the existence of the heterogeneous population of progenitors (Krzemień *et al.* 2010; Kalamarz *et al.* 2012; Ferguson and Martinez-Agosto 2017), the existence of two progenitor subpopulations at third instar has only recently been confirmed (Baldeosingh *et al.* 2018). The current study, via our analysis of various molecular markers throughout development, confirms the heterogeneity of the developing hematopoietic progenitors in the lymph gland. Thus, our study not only clarifies the cell cycle status of the *Drosophila* hematopoietic progenitors, but it is also relevant for understanding the involvement of different signaling pathways in driving the progenitors of different developmental hierarchies toward differentiation (naive to primed, primed to IP cells, and IP cells to differentiated cells).

**Figure 8:**
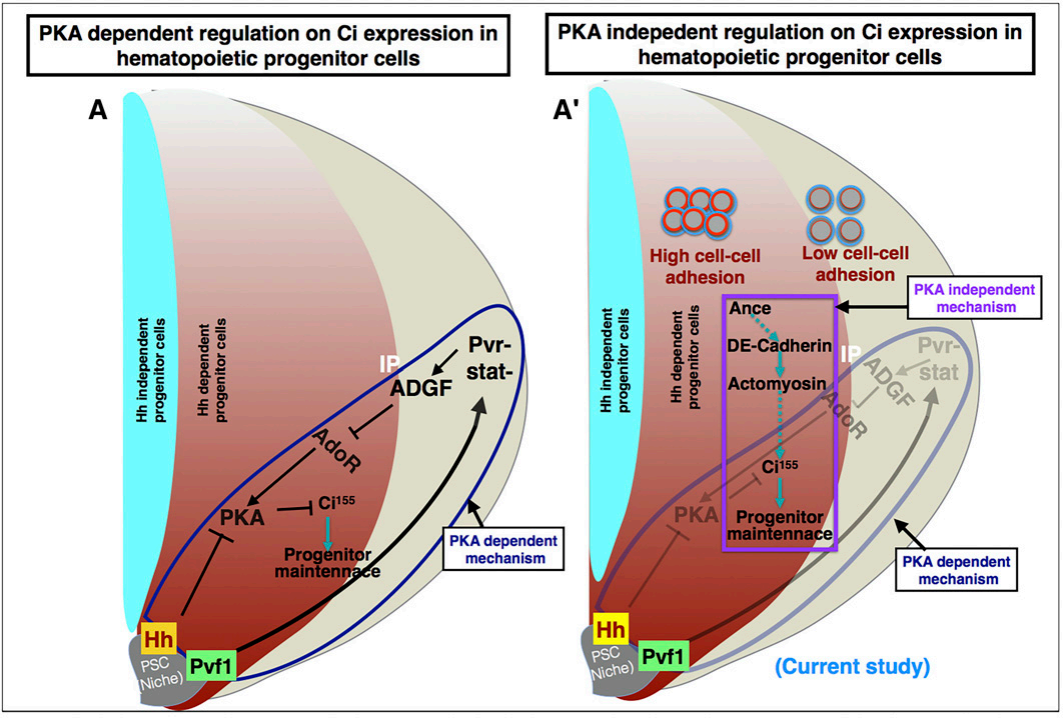
A scheme based on our results: (A) Based on the recent findings, the MZ of the late larval lymph gland consists of two distinct domains. While one domain (cayenne) is sensitive to Hh signals received from the PSC/niche, the other domain (cyan), which is juxtaposed on the dorsal vessel, is Hh-insensitive. Within the Hh-sensitive domain, two distinct pathways primarily regulate the maintenance of Ci expression in the MZ. Hh signaling from the niche and ADGF signaling from the differentiated cells enhance full-length Ci (Ci-155) expression to support progenitor maintenance in a PKA-dependent manner. (A’) We demonstrate a third axis of Ci regulation independent of PKA coupled with cell adhesion and the activity of the actomyosin network essential for progenitor homeostasis. Here, cell–cell adhesion and the actomyosin network regulate morphogen-dependent patterning of the developing lymph gland progenitors of *Drosophila* via Ci-155 activity. This combination defines the tight zonation that evolves within the developing lymph gland and is instrumental in setting up of the hierarchy of progenitors at different levels of maturation. Loss of any one component Shg, Sqh, or Zip leads to initiation of the differentiation program. Ance is a genetic interactor of this pathway described. Dotted arrows indicate mechanisms yet to be understood.

The current understanding is that the MZ of the late larval lymph gland consists of two distinct domains. While one domain is sensitive to Hh signals received from the PSC/niche, the other domain, which is juxtaposed on the dorsal vessel, is Hh-insensitive (Baldeosingh *et al.* 2018; Banerjee *et al.* 2019). Two distinct pathways primarily regulate the maintenance of Ci expression in the Hh-sensitive domain of the MZ. Hh signaling from the niche and ADGF signaling from the differentiated cells enhance Ci-155 expression to support progenitor maintenance in a PKA-dependent manner (Figure 8A).

Here, we elucidated a third axis of Ci regulation that is independent of PKA and coupled with cell adhesion and the high actomyosin activity seen in the progenitors (Figure 8B). The change in the assembly of cortical actomyosin network induced by DE-cadherin modulates Ci activity, thereby determining the progenitor status. An elegant study (Ruel *et al.* 2003) demonstrated that a scaffold complex consisting of Cos2-Fu-Ci must be translocated to the plasma membrane upon which Ci activation takes place following receipt of the Hh signal. We propose that this complex is supported by the actomyosin meshwork near the plasma membrane to facilitate Ci stabilization and activation. Therefore, loss of both cell–cell adhesion and the downstream actomyosin network results in desensitization of the hemocyte progenitors to Hh signaling, thus leading to their differentiation.

Studies of mammalian and *Drosophila* tissues have demonstrated that Hh signaling can also positively regulate cell adhesion (Jarov *et al.* 2003; Schlichting *et al.* 2005) as well as negatively (Lai *et al.* 2017). Similar to the process of vertebrate neural tube patterning, in which cadherin 7 enhances SHH signaling (Kawano *et al.* 2017), cell adhesion or the activity of the actomyosin network could regulate the activation of Hh signaling in the hemocyte progenitors of the lymph gland.

Interestingly, our search for a genetic modifier of this network revealed Ance as a candidate. Although how Ance interacts with Shg or actomyosin mechanistically is not clear, our study establishes a strong genetic link between Ance (the *Drosophila* ACE homolog), DE-cadherin and the actomyosin network that is crucial for progenitor maintenance. The conservation of this enzyme throughout the evolutionary tree indicates that it has specific functions beyond the robust RAAS (Fournier *et al.* 2012). Studies in mouse and *Drosophila* have also revealed that ACE alone is responsible for male fertility (Krege *et al.* 1995; Fuchs *et al.* 2005; Lambert *et al.* 2010), while the other RAAS components are not required (Hagaman *et al.* 1998). Furthermore, and quite strikingly, ACE can act on target genes independent of its well-known peptidase activity hinting that its first function might have been as a signaling molecule and that its peptidase activity (Fuchs *et al.* 2004; Fujihara *et al.* 2013) is a moonlighting function acquired during evolution. We believe that this study will be a starting point for using *Drosophila* as a model to tease out the RAAS-independent role of ACE in blood progenitors at the molecular level.

The formation of well-defined patterns during development requires that cells recognize their position within tissues and then differentiate accordingly. The classical concept of tissue patterning is mainly based on positional information and reaction–diffusion models (Green and Sharpe 2015; Wolpert 2016). However, more recently, cytoskeletal forces, cell adhesion, polarity, and tissue-scale mechanical signals have emerged as crucial players in tissue patterning (Meilhac *et al.* 2009; Howard *et al.* 2011; Goehring and Grill 2013, Xiong *et al.* 2014; Heller and Fuchs 2015; Chan *et al.* 2017; Gilmour *et al.* 2017). These factors alone or in various combinations can provide positional information and govern gene expression and fate at the cellular level, ultimately permitting the cells to coordinate patterning (Chan *et al.* 2017).

In the current study, we used a genetic approach to illustrate how cell–cell adhesion and the actomyosin network regulate morphogen-dependent patterning in the developing lymph gland progenitors in *Drosophila*. The cooperation between these factors defines the tight zonation that evolves within the developing lymph gland to establish a hierarchy in the progenitors at different stages of maturation.
